# Matrix Gla protein polymorphism rs1800802 is associated with atheroma plaque progression and with cardiovascular events in a chronic kidney disease cohort

**DOI:** 10.1093/ckj/sfad257

**Published:** 2023-10-10

**Authors:** Serafí Cambray, Marcelino Bermúdez-López, Alicia Garcia-Carrasco, Jose M Valdivielso, NEFRONA investigators, Mª José Aladrén Regidor, Jaume Almirall, Esther Ponz, Jesús Arteaga Coloma, Bajo Rubio, Mª José Aladrén Regidor, Belart Rodríguez, Antonio Gascón, Jordi Bover Sanjuan, Josep Bronsoms Artero, Juan B Cabezuelo Romero, Muray Cases, Jesús Calviño Varela, Pilar Caro Acevedo, Jordi Carreras Bassa, Aleix Cases Amenós, Elisabet Massó Jiménez, Rosario Moreno López, Secundino Cigarrán Guldris, Saray López Prieto, Lourdes Comas Mongay, Isabel Comerma, Mª Teresa Compte Jové, Marta Cuberes Izquierdo, Fernando de Álvaro, Covadonga Hevia Ojanguren, Gabriel de Arriba de la Fuente, Mª Dolores del Pino y Pino, Rafael Diaz-Tejeiro Izquierdo, Marta Dotori, Verónica Duarte, Sara Estupiñan Torres, Mª José Fernández Reyes, Mª Loreto Fernández Rodríguez, Guillermina Fernández, Antonio Galán Serrano, Cesar García Cantón, Antonio L García Herrera, Mercedes García Mena, Luis Gil Sacaluga, José Luis Górriz, Emma Huarte Loza, José Luis Lerma, Antonio Liebana Cañada, Jesús Pedro Marín Álvarez, Nàdia Martín Alemany, Jesús Martín García, Alberto Martínez Castelao, María Martínez Villaescusa, Isabel Martínez, Iñigo Moina Eguren, Silvia Moreno Los Huertos, Ricardo Mouzo Mirco, Antonia Munar Vila, Ana Beatriz Muñoz Díaz, Juan F Navarro González, Javier Nieto, Agustín Carreño, Enrique Novoa Fernández, Alberto Ortiz, Vicente Paraíso, Miguel Pérez Fontán, Ana Peris Domingo, Celestino Piñera Haces, Mª Dolores Prados Garrido, Mario Prieto Velasco, Carmina Puig Marí, Maite Rivera Gorrín, Esther Rubio, Pilar Ruiz, Mercedes Salgueira Lazo, Ana Isabel Martínez Puerto, José Antonio Sánchez Tomero, José Emilio Sánchez, Ramon Sans Lorman, Ramon Saracho, Maria Sarrias, Daniel Serón, María José Soler, Clara Barrios, Fernando Sousa, Daniel Toran, Fernando Tornero Molina, José Javier Usón Carrasco, Ildefonso Valera Cortes, Mª Merce Vilaprinyo del Perugia, Rafael C Virto Ruiz, Inés GilGil Área Básica Sanitaria de Arán, Jose Mª Fernández Toro, Juan Antonio Divisón Garrote Centro de Salud de Casas Ibáñez

**Affiliations:** Vascular and Renal Translational Research Group, Biomedical Research Institute of Lleida Fundació Dr Pifarré (IRBLleida ); Department of Basic Medical Sciences, University of Lleida, Lleida, Spain; Vascular and Renal Translational Research Group, Biomedical Research Institute of Lleida Fundació Dr Pifarré (IRBLleida ); Department of Experimental Medicine, University of Lleida, Lleida, Spain; Vascular and Renal Translational Research Group, Biomedical Research Institute of Lleida Fundació Dr Pifarré (IRBLleida ); Vascular and Renal Translational Research Group, Biomedical Research Institute of Lleida Fundació Dr Pifarré (IRBLleida )

**Keywords:** atherosclerosis progression, cardiovascular events, chronic kidney disease, matrix gamma-carboxy glutamic acid (Gla) protein, polymorphisms

## Abstract

**Background:**

Chronic kidney disease (CKD) is associated with increased atherosclerotic burden and higher risk for cardiovascular events (CVE). Atherosclerosis has a significant genetic component and, in CKD, it is influenced by mineral metabolism alterations. Therefore, genetic modifications of mineral metabolism–related proteins could affect atherosclerosis in CKD patients. In the present study we investigated the role of single nucleotide polymorphisms (SNPs) of the matrix gamma-carboxy glutamic acid protein (MGP) on atherosclerosis progression and CVE in a CKD cohort.

**Methods:**

A total of 2187 CKD patients from the Observatorio Nacional de Aterosclerosis en Nefrologia (NEFRONA) study were genotyped for SNPs present in the *matrix gamma-carboxy glutamic acid (Gla) protein* (*MGP*) gene. Atheromatosis was detected by vascular ultrasound. Progression of atheromatosis, defined as an increase in territories with plaque, was assessed after 24 months. Patients were followed for 48 months for CVE. Association of SNPs with plaque progression was assessed by logistic regression and their capacity to predict CVE by Cox regression.

**Results:**

Three SNPs of the *MGP* gene were analyzed. No association of the rs4236 or the rs1800801 SNPs was detected with any of the outcomes. However, patients homozygotes for the minor allele of the rs1800802 SNP showed higher adjusted risk for plaque progression [odds ratio 2.3 (95% confidence interval 1.06–4.9)] and higher risk of suffering a CVE [hazard ratio 2.16 (95% confidence interval 1.13–4.12)] compared with the rest of genotypes. No association of the SNP with total or dp-ucMGP levels was found in a subsample.

**Conclusions:**

The rs1800802 polymorphism of MGP is associated with plaque progression and CVE in CKD patients.

## Graphical Abstract

**Graphical Abstract:**
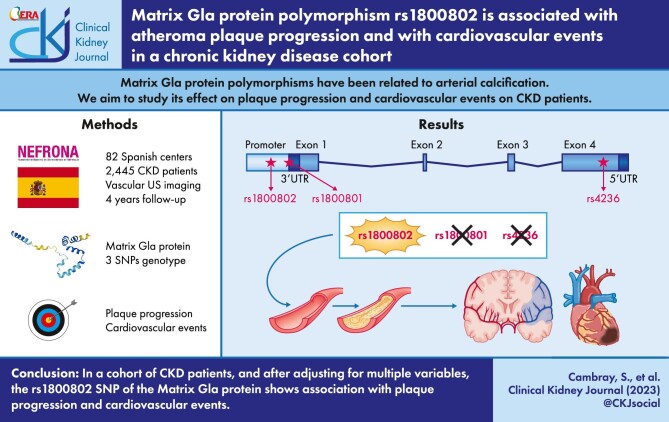

KEY LEARNING POINTS **What was known:**

- Serum levels of matrix gamma-carboxy glutamic acid protein (MGP) are related to arterial calcification and cardiovascular events (CVE), particularly in chronic kidney disease (CKD) patients.
- The correlation of single nucleotide polymorphisms (SNPs) in the *MGP* gene with these outcomes varies depending on genetic background or CKD stage, and furthermore, investigations have mostly focused on coronary calcifications.
- Our study employs a large prospective Spanish cohort [Observatorio Nacional de Aterosclerosis en Nefrologia (NEFRONA)] with different CKD stages over a 4-year period to examine the effects of these polymorphisms on arterial calcification while controlling for multiple parameters and confounding factors.

**This study adds:**

- Even after controlling for several confounding factors, we report that the rs1800802 SNP has an independent connection with arterial atheromatous plaque progression after 2 years, and with CVE after 4 years in CKD patients.
- This is the first time an SNP found in a CKD population has been linked to both arterial atheromatosis progression and CVE.

**Potential impact:**

- The relationship of the rs1800802 SNP with plaque progression and CVE provides a helpful tool for improving risk stratification and to understand atherosclerosis progression in the CKD population, paving the way for personalized prognosis and treatment in this population.

## INTRODUCTION

According to the Global Burden of Disease Study, chronic kidney disease (CKD) is among the top causes of disability-adjusted life-years (DALYs); in 1990 it was the 29th cause, but in 2019 the DALYs caused by CKD doubled, climbing to the 18th position. When considering the population over 50 years of age, CKD is the 8th contributor to DALYs, also showing a 100% increase in the number of DALYs caused compared with 1990 [1]. This pattern correlates with the prediction on number of years of life lost (YLLs). Thus, in 2016 CKD was the 16th leading cause, and it is predicted that in 2040 CKD will become the 5th cause of YLLs [2]. The main cause of death among CKD patients is of cardiovascular origin due to their higher risk for cardiovascular events (CVE) [3] and its associated increase in cardiovascular deaths (CVD) [4], both related to the higher prevalence of atherosclerosis in these patients [5]. For this reason many efforts have been focused on identifying atherosclerotic biomarkers in order to predict CVE and CVD [6], among which proteins involved in mineral metabolism have been widely analyzed [7]. One of these proteins, matrix gamma-carboxy glutamic acid (Gla) protein (MGP), an extracellular vitamin K–dependent protein, plays an essential role in arterial calcification [8].

MGP was initially described as a powerful inhibitor of arterial calcification in mice [9]. Later studies showed that MGP serum levels in humans are influencing arterial calcification and cardiovascular disease [8], particularly in CKD patients [10, 11] in which they also associate with all-cause and cardiovascular mortality [12]. Eight single nucleotide polymorphisms (SNPs) in the coding and 5′-flanking sequences of the *MGP* gene were discovered by Cambien's lab in groundbreaking research demonstrating that two of them confer an increased risk for plaque calcification and myocardial infarction. In addition, one of these SNPs was related to decreased MGP expression [13]. Since then, a number of other studies have concentrated on SNPs in the *MGP* gene and their relationship to arterial calcification and cardiovascular diseases [14–16], some of them in CKD populations [11, 17]. Current studies are focused on three of the SNPs [rs1800802 (T138-C), rs1800801 (G7-A) and rs4236 (Thr83-Ala)], due to the lack of association of the others with arterial calcification. However, results regarding its association with vascular calcification and/or CVE are still controversial, showing different results depending upon the genetic background and the disease of study [18].

In light of these findings, we looked into the relationship between the SNPs rs1800802, rs1800801 and rs4336 and the development of atherosclerotic plaque and CVE in a group of CKD patients during a 48-month period.

## MATERIALS AND METHODS

### Study population

The Observatorio Nacional de Aterosclerosis en Nefrologia (NEFRONA) project is a prospective, multicenter observational cohort study with the goal of examining the prognostic significance of ultrasonography data and serological and genetic biomarkers in CVE and CVD in Spanish CKD patients [19]. The design of the study has been published previously [20]. Briefly, 2445 CKD patients (937 in CKD G3, 820 in CKD G4-5 and 688 in CKD G5D) and 559 controls with normal renal function, with ages between 18 and 75 years, were enrolled between 2009 and 2012. All patients were free from a previous CVE and were monitored for 4 years to collect information on CVE, CVD, non-CVD or the start of renal replacement therapy. The clinicians in charge of patient recruitment used the International Classification of Diseases, Ninth Revision, Clinical Modification to determine the incidence of CVEs. Causes of death outside the hospital settings were determined by interviewing the patient's family. Each participant signed an informed consent form. The local ethics committee of each hospital approved the study. The following conditions were included in the exclusion criteria: history of CVEs, severe carotid stenosis, active infections (tuberculosis, hepatitis), pregnancy and life expectancy <12 months.

We selected CKD patients with Caucasian background from the NEFRONA study with data on the analyzed SNPs (2187; 72.8% of the NEFRONA study participants). To study the relationship of the genotyped SNPs of the *MGP* gene with atheroma plaque progression and incidence of CVE, we elaborated models of dominance and codominance.

B-mode ultrasound of the carotid and femoral arteries was performed in 10 arterial territories using the Vivid apparatus (General Electric) equipped with a 6–13 MHz broadband linear array probe as previously described [21]. Briefly, ultrasound imaging was performed with the subjects in a supine position and the head turned 45° contralateral to the side of the probe to evaluate carotid plaques. The presence of atheromatous plaques was defined as a intima-media thickness (IMT) >1.5 mm protruding into the lumen, according to the American Society of Echocardiography Consensus Statement [22] and the Mannheim IMT Consensus [23]. Plaque progression was defined as an increase in the number of territories with plaque in a second ultrasound study, 24 months after the first evaluation.

### SNP genotyping

The biobank of the Spanish Renal Research Network (REDinREN) [24] provided DNA from stored blood samples, which was extracted using the QIAamp DNA Blood Kit following manufacturer instructions. A matrix-assisted laser desorption ionization time-of-flight mass spectrometry in the Sequenom MassARRAY platform^®^ was used for genotyping in the Centro de Genotipado-Plataforma de Recursos Biomoleculares y Bioinformáticos (CEGEN-PRB2) del Instituto de Salud Carlos III (Nodo de la Universidad de Santiago de Compostela, A Coruña, Spain). SNP clusters, samples with low genotyping percentage and SNPs not meeting the Hardy–Weinberg equilibrium (HWE) were eliminated. Replicates of samples and samples from the Coriell Institute Biorepository were included to ensure genotyping quality. Current study focuses on three SNPs of the *MGP*, two of them located in the 5′-flanking sequence of the *MGP* gene (rs1800801 and rs1800802) and one located in exon 4 (rs45236).

### Plasma MGP measurement

Venous blood samples were collected in citrate tubes and plasma was isolated and stored at –80°C until use. To measure total MGP in plasma we used the Human MGP ELISA Kit (Cat. No. EH1649; Fine Test; Wuhan Fine Biotech Co., Ltd, China). Intra-assay coefficients of variation is <8%, and inter-assay coefficient of variation is <10%. To measure dephosphorylated uncarboxylated MGP (dp-ucMGP) in plasma we used the Human dp-ucMGP ELISA Kit (Cat. No. EH4755; Fine Test; Wuhan Fine Biotech Co., Ltd, China). Intra-assay coefficients of variation is <8%, and inter-assay coefficient of variation is <10%.

### Statistical analysis

For qualitative variables, number and percentage is shown; for quantitative variables median and first–third quartile, or mean and standard deviation is reported, depending on normality of distribution. Comparisons between non-normally distributed quantitative variables were performed with Mann–Whitney U test, and with Student's *t*-test for normally distributed ones; statistically significant differences between categorical data were analyzed with Chi-squared test. The HWE was calculated using Gene-Calc, which compares observed and expected genotype frequencies using the Chi-square goodness-of-fit test [25]. All the statistical analysis were done using SPSS statistics software version 25 (IBM, Armonk, NY, USA).

## RESULTS

Table 1 shows no association of rs1800801 and rs4236 with plaque progression or CVE in any of the proposed models, but the rs1800802 polymorphism was associated with both outcomes in the two models (dominant and co-dominant). For further analysis, we considered the dominant model in which patients homozygous for the less frequent CC genotype showed more severe plaque progression and more CVE when compared with patients with the TT or CT genotypes. To study the association of the rs1800802 genotypes with all the cardiovascular risk factors registered, we conducted a bivariate analysis. As seen in Table 2, patients homozygote for the C allele showed statistically significant lower levels of diastolic blood pressure, low-density lipoprotein (LDL) cholesterol, calcium and sodium.

**Table 1:** Association of the three MGP SNPs to plaque progression and CVE.

			Plaque progression				CVE		
			No progression,	1–2 plaques,	≥3 plaques,		No,	Yes,	
SNP (*n*)	Model	Genotype	*n* (%)	*n* (%)	*n* = 212	*P*-value	*n* (%)	*n* (%)	*P*-value
rs4236 (2186)	Dominant	AA + AG	487 (39.9)	562 (46)	172 (14.1)	0.945	1645 (91.7)	149 (8.3)	0.373
		GG	106 (39.4)	123 (45.7)	40 (14.9)		354 (90.3)	38 (9.7)	
	Co-dominant	AA	187 (38.8)	217 (45)	78 (16.2)	0.561	633 (91.2)	61 (8.8)	0.568
		AG	300 (40.6)	345 (46.7)	94 (12.7)		1012 (92)	88 (8)	
		GG	106 (39.4)	123 (45.7)	40 (14.9)		354 (90.3)	38 (9.7)	
	HWE (*P*-value)		0.751	0.784	0.475		0.345	0.831	
rs1800802 (2187)	Dominant	TT-CT	582 (40.3)	665 (46.1)	196 (13.6)	.000283	1943 (91.7)	176 (8.3)	.007
		CC	11 (22.9)	21 (44.6)	16 (33.3)		56 (82.4)	12 (17.6)	
	Co-dominant	TT	413 (41.5)	443 (44.5)	139 (14)	.001	1355 (91.6)	125 (8.4)	.024
		CT	169 (37.7)	222 (49.6)	57 (12.7)		588 (92)	51 (8)	
		CC	11 (22.9)	21 (44.6)	16 (33.3)		56 (82.4)	12 (17.6)	
	HWE (*P*-value)		0.412	0.557	0.022		0.715	0.118	
rs1800801 (2187)	Dominant	GG + GA	496 (39.8)	575 (46.1)	175 (14)	.864	1679 (91.4)	157 (8.6)	.864
		AA	97 (39.6)	111 (45.3)	37 (15.1)		320 (91.2)	31 (8.8)	
	Co-dominant	GG	209 (38.8)	239 (44.4)	90 (16.7)	.213	711 (91.3)	68 (8.7)	.959
		GA	287 (40.5)	336 (47.5)	85 (12)		968 (91.6)	89 (8.4)	
		AA	97 (39.6)	111 (45.3)	37 (15.1)		320 (91.2)	31 (8.8)	
	HWE (*P*-value)		0.9958	0.926	0.108		0.952	0.978	

**Table 2:** Association of MGP rs1800802 T/C polymorphism to epidemiological, clinical and biochemical parameters of the cohort.

Variable	Homozygote TT + heterocygote CT, *n* = 2119	Homozygote CC, *n* = 68	*P*-value
Age (years)	62 (50; 68)	60 (51.5; 69)	.685
Sex (female)	801 (37.8)	24 (35.5)	.675
Smoking	1210 (57.1)	43 (63.2)	.314
Diabetes	535 (25.2)	20 (29.4)	.437
Hypertension	1930 (91.1)	62 (91.2)	.978
Dyslipemia	1419 (67)	48 (70.6)	.531
CKD stage			
G3	839 (39.6)	28 (41.2)	.084
G4–5	709 (33.5)	15 (22.1)	
G5D	571 (26.9)	25 (36.1)	
Body mass index (kg/m^2^)	27.76 (24.8; 31.3)	27.8 (24; 31)	.614
Systolic blood pressure (mmHg)	141 (128; 157)	139 (125; 155)	.158
Diastolic blood pressure (mmHg)	81 (74; 89)	78.5 (70; 86.5)	.023
Pulse pressure (mmHg)	59 (49; 72)	56 (49; 72)	.489
Total cholesterol (mg/dL)	175 (152; 202)	168 (142; 202)	.217
HDL cholesterol (mg/dL)	47 (38; 58)	46 (37; 53)	.103
LDL cholesterol (mg/dL)	101 (78; 122)	89.5 (73; 110)	.03
Triglycerides (mg/dL)	125 (92; 173)	136 (98; 185)	.321
Glucose (mg/dL)	97 (87; 113)	98 (87; 130)	.304
Calcium (mg/dL)	9.3 (9; 9.7)	9.2 (8.8; 9.5)	.012
Phosphate (mg/dL)	3.8 (3.3; 4.6)	3.8 (3.3; 4.4)	.782
Sodium (mEq/L)	140.1 (139; 142)	140 (137; 142)	.037
Potassium (mEq/L)	4.8 (4.4; 5.2)	4.8 (4.4; 5.3)	.609
25-OH vitamin D (ng/mL)	15 (11.2; 19.2)	14.75 (10.8; 19.3)	.858
Plaque at baseline	1489 (70.7)	53 (77.9)	.195
Plaque progression			
No progression	582 (40.3)	11 (22.9)	.000
1–2 plaques	665 (46.1)	21 (43.8)	
≥3 plaques	196 (13.6)	16 (33.3)	
Cardiovascular event	176 (8.3)	12 (17.6)	.007

Qualitative variables are expressed as *N* (%). Quantitative variables are expressed as median (Q1; Q3). Comparisons between groups was performed with Mann–Whitney U test for quantitative variables and Chi-squared test for categorical data.

To further analyze the relationship of severe plaque progression (≥3 new plaques on the 24-month follow-up) with the CC genotype, we stratified the cohort according to plaque progression, and compared cardiovascular risk factors among different groups. As shown in Table 3, severe plaque progression (≥3 new plaques) was linked to increased age, body mass index, systolic blood pressure, pulse pressure, glucose, potassium and 25-hydroxy (25-OH) vitamin D levels. Showing new plaque in ≥3 arterial territories on the follow-up was also associated with sex, smoking, diabetes, hypertension, dyslipemia, presenting with ≥1 plaque on basal evaluation and with exhibiting the CC alleles of the rs18002802 polymorphism. We performed two prognostic logistic regression models, one considering progression the appearance of ≥1 new plaque in the follow up, and the other considering only severe progression (presence of ≥3 new plaques) (Table 4). Age, smoking, being in dialysis and potassium levels predicted both, the presence of ≥1 new plaques or the presence of ≥3 new plaques. Having plaque already at basal examination was also a predictor of the appearance of ≥1 new plaque at follow-up. Finally, being homozygote for less frequent allele of the rs18002802 polymorphism was predictive of both conditions.

**Table 3:** Epidemiological, clinical and biochemical parameters according to plaque progression.

Variable	No progression, *n* = 593	1–2 plaques, *n* = 686	≥3 plaques, *n* = 212	*P*-value
Age (years)	58 (43.5; 66)	63 (54; 69)	67 (60; 70)	.000
Sex (female)	253 (42.7)	240 (35)	61 (28)	.000
Smoking	297 (50.1)	415 (60.5)	143 (67.5)	.000
Diabetes	104 (17.5)	197 (28.7))	72 (34)	.000
Hypertension	529 (89.2)	642 (93.6)	203 (95.8)	.002
Dyslipemia (yes)	378 (63.7)	501 (73)	155 (73)	.001
CKD Stage				
G3	274 (46.2)	316 (46.1)	79 (37.3)	.073
G4–5	219 (36.9)	242 (35.3)	81 (38.2)	
G5D	100 (16.9)	128 (18.7)	52 (24)	
Body mass index (kg/m^2^)	27.7 (24.6; 31.5)	28.3 (25; 32)	28.7 (25.7; 32)	.024^c^
Systolic blood pressure (mmHg)	138 (126; 151)	142 (130; 158)	146 (132; 162)	.000^d^
Diastolic blood pressure (mmHg)	81 (75; 88)	81 (74: 89)	80 (73; 88)	.794
Pulse pressure (mmHg)	55 (46; 67)	60 (50; 73.2)	65 (53; 78)	.000^a^
Total cholesterol (mg/dL)	179 (156; 203)	176 (153; 206)	175 (148; 202)	.321
HDL cholesterol (mg/dL)	48 (39; 58)	47 (38; 58)	46 (39; 55)	.342
LDL cholesterol (mg/dL)	103 (82.6; 123)	101 (79; 120)	101 (78; 123)	.421
Triglycerides (mg/dL)	118 (88; 164)	126.5 (94; 178)	133 (99; 170)	.04^b^
Glucose (mg/dL)	95 (86; 107)	99 (89; 117)	101 (90; 123)	.007^d^
Calcium (mg/dL)	9.39 (9; 9.7)	9.4 (9; 9.7)	9.3 (8.9; 9.6)	.072
Phosphate (mg/dL)	3.7 (3.2; 4.3)	3.7 (3.3; 4.4)	3.8 (3.4; 4.5)	.597
Sodium (mEq/L)	140.6 (139; 142)	141 (139; 142)	141 (139; 142)	.818
Potassium (mEq/L)	4.7 (4.3; 5.1)	4.8 (4.5; 5.1)	4.9 (4.5; 5.3)	.003^e^
25-OH vitamin D (ng/mL)	15.9 (12.2; 20.6)	15.1 (11.3; 19)	13.8 (10.5; 17.8)	.001^d^
Plaque at baseline	319 (53.8)	531 (77.4)	185 (87.3)	.000
rs1800802				
TT + TC	582 (98.1)	665 (96.9)	196 (92.5)	.000
CC	11 (1.9)	21 (3.1)	16 (7.5)	

Qualitative variables are expressed as *N* (%). Quantitative variables are expressed as median (Q1; Q3). Comparisons between groups performed with Kruskal–Wallis test for quantitative variables, difference among groups was calculated with pairwise comparisons. Chi-squared test was used for categorical data.

^a^Difference among all groups.

^b^difference between no plaque progression and 1 or 2 new plaques.

^c^difference between no plaque progression and ≥3 new plaques.

^d^difference between no plaque progression and the other groups.

^e^difference between ≥3 new plaques and the other groups.

**Table 4:** Logistic regression to model plaque progression.

	≥1 new plaque (no, 515; yes, 791)		≥3 new plaques (no, 1118; yes, 188)	
Variable	OR (95% CI)	*P*-value	OR (95% CI)	*P*-value
Age	1.043 (1.030–1.057)	.000	1.059 (1.03–1.08)	.000
Smoking	1.7 (1.3–2.23)	.000	1.66 (1.1–2.48)	.014
Any plaque basal	1.64 (1.22–2.21)	.001		
Potassium	1.33 (1.07–1.66)	.011	1.34 (1.01–1.79)	.04
CKD G4–5 (vs G3)	1.035 (0.78–1.36)	.806	1.44 (0.99–2.1)	.055
CKD G5D (vs G3)	1.91 (1.32–2.76)	.001	2.105 (1.31–3.37)	.002
rs1800802 (CC)	2.35 (1.04–5.3)	.039	3.62 (1.75–7.48)	.000

Results are expressed as odds ratios (OR) and 95% confidence interval (95% CI).

Variables that did not reach statistical significance: sex, diabetes, hypertension, dyslipemia, body mass index, systolic blood pressure, pulse pressure, triglycerides, glucose, HDL cholesterol and 25-OH vitamin D.

Another interesting association of the rs18002802 polymorphism was with the incidence of a CVE after 48 months of follow-up. Table 5 shows the epidemiological, clinical and biochemical parameters of our cohort according with the presence of CVE. A total of 188 of the participants suffered a CVE (8.6% of the selected participants). The individuals showing a CVE were mainly males, smokers, diabetics and dyslipidemic, and had plaque presence at baseline. Furthermore, they displayed older age and increased body mass index, systolic blood pressure, pulse pressure, triglycerides, glucose, phosphate and potassium. They also presented with lower high-density lipoprotein (HDL) cholesterol, sodium and vitamin D levels (Table 5).

**Table 5:** Epidemiological, clinical and biochemical parameters of the cohort according to the incidence of CVEs.

Variable	No CVE, *n* = 1999	CVE, *n* = 188	*P*-value
Age (years)	61 (50; 68)	64.5 (58; 70)	.000
Sex (female),	771 (38.6)	54 (28.7)	.008
Smoking	1129 (56.5)	124 (66)	.012
Diabetes	472 (23.6)	83 (44.1)	.000
Hypertension	1814 (90.7)	178 (94.7)	.07
Dyslipemia	1328 (66.4)	139 (73.9)	.036
CKD Stage			
G3	802 (40.1)	65 (34.6)	.185
G4–5	662 (33.1)	62 (33)	
G5D	535 (26.8)	61 (32.4)	
Body mass index (kg/m^2^)	27.6 (24.68; 31.2)	29 (25.8; 32.2)	.006
Systolic blood pressure (mmHg)	140 (128; 156)	149 (132; 164)	.000
Diastolic blood pressure (mmHg)	81 (74; 89)	81 (71; 89)	.705
Pulse pressure (mmHg)	59 (48; 71)	68 (54; 80)	.000
Total cholesterol (mg/dL)	176 (152; 202)	170.5 (141.2; 206)	.277
HDL cholesterol (mg/dL)	47 (39; 58)	44 (34; 53)	.001
LDL cholesterol (mg/dL)	101 (79; 121)	98 (72; 124)	.447
Triglycerides (mg/dL)	124 (91; 172)	141 (103; 183)	.006
Glucose (mg/dL)	96 (87; 111)	105 (91.2; 146.7)	.000
Calcium (mg/dL)	9.3 (9; 9.7)	9.3 (8.9; 9.7)	.321
Phosphate (mg/dL)	3.8 (3.3; 4.5)	4 (3.4; 5)	.017
Sodium (mEq/L)	140.1 (139; 142)	140 (138; 142)	.016
Potassium (mEq/L)	4.8 (4.4; 5.2)	4.9 (4.5; 5.3)	.002
25-OH vitamin D (ng/mL)	15.19 (11.4; 19.5)	13.15 (9.6; 17.2)	.000
Plaque at baseline	1377 (68.9)	174 (92.6)	.000
rs1800802 (CC)	56 (82.4)	12 (17.6)	.000

Qualitative variables are expressed as *N* (%). Quantitative variables are expressed as median (Q1; Q3).

Comparisons between groups was performed with Mann–Whitney U test for quantitative variables and Chi-squared test for categorical data.

**Figure 1:**
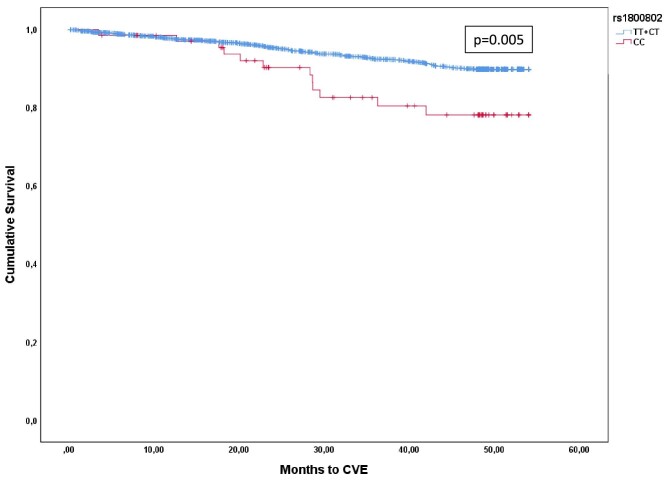
Kaplan–Meier curves showing the incidence of cardiovascular events depending on genotype during the follow-up period. Log-rank test comparing the survival distributions showed significant differences (*P*-value = 0.005).

Next, we conducted a Kaplan–Meier survival analysis to better assess the impact of the rs1800802 on CVE. Figure 1 demonstrates that compared with patients with the other two genotypes, patients homozygous for the less dominant allele have a higher frequency of CVE (*P*-value .005).

Finally, to assess the importance of the rs1800802 SNP in predicting CVE, we performed a Cox regression analysis adjusting for all the variables that could contribute to CVE. Table 6 shows that having plaque at baseline is a strong predictor of future CVE. Other variables showing association with CVE are older age, being diabetic and higher levels of HDL cholesterol and 25-OH vitamin D, together with lower levels of phosphate. Interestingly, sex did not reach significant association with future CVE. The rs1800802 polymorphism still showed a significant association with CVE incidence. Plasma levels of total MGP and dp-ucMGP measured in a subsample of the study (*n* = 80) were not associated with the rs1800802 SNP (Supplementary data, Tables S1 and S2). Total MGP levels, but not dp-ucMGP, were associated with CVE (Supplementary data, Tables S3 and S4).

**Table 6:** Cox regression modelling the incidence of CVEs.

Variable	HR (95% CI)	*P*-value
Sex	0.77 (0.54–1.1)	.16
Age	1.02 (1–1.03)	.062
Diabetes	1.6 (1.16–2.2)	.004
Plaque at baseline	3.1 (1.64–5.8)	.000
HDL cholesterol	0.98 (0.97–1)	.022
Phosphate	1.4 (1.22–1.58)	.000
25-OH vitamin D	0.94 (0.92–0.97)	.000
rs1800802 (CC)	2.16 (1.13–4.12)	.019

Results are expressed as hazard ratio (HR) and 95% confidence interval (95% CI).

Variables that did not reach significance level: sex, smoker, dyslipidemia, body mass index, systolic blood pressure, diastolic blood pressure, triglycerides, glucose, sodium and potassium.

## DISCUSSION

In the current study we have shown that there is an independent association of the rs1800802 SNP of the MGP gene with CVE after 4 years, and with atheromatous plaque progression after 2 years in CKD patients. Indeed, previous studies in the NEFRONA cohort have shown an increased prevalence and progression of atherosclerosis in patients with CKD, even at early stages [26].

The rs1800802 polymorphism is located at the promoter of the *MGP* gene that codes for the matrix Gla protein, a vitamin K–dependent protein that plays a crucial role in arterial calcification [27]. A first study described that the minor T138C allele reduced the promoter activity of the *MGP* gene [13]. A year later, another study showed opposite effects using a primary cell culture of vascular smooth muscle cells. Thus, Farzaneh-Far *et al*. showed that the rs1800802 SNP interfered with the binding of the AP-1 transcription factor in the *MGP* promoter, altering the expression of the gene and increasing circulating levels of the protein [28]. In our study we did not find any correlation of rs1800802 with either plasma levels of MGP or with dp-ucMGP, but due to the small sample size used for this analysis our result must be interpreted cautiously.

MGP is a calcification inhibitor that is highly expressed in kidney [29], which requires a carboxylation and a phosphorylation to become fully active. Lower levels of active MGP are associated with arterial calcification, CVEs and CVD [8, 30]. In addition, circulating levels of the inactive form of MGP increase in parallel with CKD progression, showing an inverse correlation with estimated glomerular filtration rate [31].

Many studies have analyzed the effect of SNP of the *MGP* gene on arterial calcification, but most of them focusing on coronary calcifications, which is a surrogate marker of coronary atheroma plaque presence [14, 16, 32, 33]. Due to the inverse correlation of active MGP levels with CKD progression, some studies have also focused on CKD patients [11, 17, 34, 35]. Despite findings showing effects on coronary artery calcifications for the rs1800801 [13, 18, 32, 36] and the rs4236 [13, 36] polymorphisms, only one of the studies found association with the rs1800802 SNP [36]. Although carotid arteries and coronary arteries share similarities in vasomotor function and anatomical structure [37], plaque formation in both seems to be influenced by distinct genetic and biological factors [38]. Indeed, the heredity patterns of plaque presence between these two structures is essentially different [39–42]. Hence, this fact could explain the differences between the previous findings in coronary calcification and our results focused on systemic atherosclerotic plaque.

A previous study in a CKD cohort found a relationship of the rs1800801 and the rs4236 polymorphisms, but not rs1800802, with medial vascular calcification [11]. Furthermore, another study with a population that comprised mainly CKD G5D patients showed that rs1800802 was associated with being in dialysis and with suffering a CVD [34]. In previous studies, we did not found an association of the rs18008002 SNP with CKD [43], and in the present study, despite a clear tendency, there is no association of the rs1800802 CC polymorphism with presenting CVD (TT + TC: 59 CVD, 2.2% of group; CC: 4 CVD, 4.8% of group; *P*-value .122; data not shown). The discrepancies with reported CVD in Brancaccio *et al*.’s study [34] could be due to the high proportion of CKD G5D patients present in their CKD cohort (79.5%), a subset of CKD patients with a high CVD risk.

More recently, in meta-analysis evaluating the association of the rs1800802 polymorphism with the risk of vascular calcification and atherosclerotic disease analyzing nine studies (2073 cases and 2177 controls), the authors did not find significant associations in any of the genetic models [18]. It should be noted that for this meta-analysis, the authors mixed CKD [34] and non-CKD [13, 15, 44] populations, and populations with coronary artery calcifications [33, 36, 44] with populations presenting plaques in the femoral and carotid arteries [13]. Moreover, the CKD population of the meta-analysis did not provide any data on artery calcification [34], so their results cannot be extrapolated, nor compared with our cohort.

Using the NEFRONA cohort, we have previously described polymorphisms involved in vascular calcification [45], CVE [46] and atheromatosis progression [47], but the rs1800802 is the first described polymorphism from the NEFRONA cohort that is associated with both, plaque progression and CVE, even after adjusting for several confounders. Indeed, other studies have identified SNPs involved in atherosclerosis progression [48–52], but to the extent of our knowledge this is the first time that an SNP has been associated with both, atheromatosis progression and CVE.

Despite the novelty of the results, this research has several limitations. First, the lack of an independent cohort to validate our results, second is the small number of patients carrying the rs1800802-CC genotype, and the low number of plasma samples in which we were able to quantify total MGP and dp-ucMGP levels. As for the strengths of the current work, we would like highlight the prospective design of the study, and the fairly large number of CKD patients with follow-up for plaque progression and CVE. Moreover, the NEFRONA database allowed us to adjust our results for multiple parameters and confounding factors.

In conclusion, we have found that the rs1800802 SNP of the *MGP* gene is independently associated with cardiovascular events and plaque progression. Despite the need for further validation this is a promising polymorphism for use in CVE prediction in CKD patients.

## Supplementary Material

sfad257_Supplemental_File

## Data Availability

The data underlying this article will be shared on reasonable request to the corresponding author.

## Appendix

NEFRONA investigators: the NEFRONA study investigator group is composed by the following: Aladrén Regidor, Mª José. Hospital Comarcal Ernest Lluch (Calatayud); Almirall, Jaume; Ponz, Esther. Corporació Parc Taulí (Barcelona); Arteaga Coloma, Jesús. Hospital de Navarra (Pamplona); Bajo Rubio, Mª Auxiliadora; Díaz, Raquel Raquel Hospital La Paz (Madrid); Belart Rodríguez, Montserrat. Sistemes Renals (Lleida); Gascón, Antonio, Hospital Obispo Polanco (Teruel); Bover Sanjuan, Jordi. Fundació Puigvert. IIB Sant Pau (Barcelona); Bronsoms Artero, Josep. Clínica Girona (Girona); Cabezuelo Romero, Juan B; Muray Cases, Salomé. Hospital Reina Sofía (Murcia); Calviño Varela, Jesús. Hospital Universitario Lugus Augusti (Lugo); Caro Acevedo, Pilar. Clínica Ruber (Madrid); Carreras Bassa, Jordi. Diaverum Baix Llobregat (Barcelona); Cases Amenós, Aleix; Massó Jiménez, Elisabet. Hospital Clínic (Barcelona); Moreno López, Rosario. Hospital de la Defensa (Zaragoza); Cigarrán Guldris, Secundino; López Prieto, Saray. Hospital Da Costa (Lugo); Comas Mongay, Lourdes. Hospital General de Vic (Barcelona); Comerma, Isabel. Hospital General de Manresa (Barcelona); Compte Jové, Mª Teresa, Hospital Santa Creu Jesús (Tarragona); Cuberes Izquierdo, Marta. Hospital Reina Sofía (Navarra); de Álvaro, Fernando; Hevia Ojanguren, Covadonga. Hospital Infanta Sofía (Madrid); de Arriba de la Fuente, Gabriel. Hospital Universitario Guadalajara (Guadalajara); del Pino y Pino, Mª Dolores. Complejo Hospitalario Universitario Torrecardenas (Almería); Diaz-Tejeiro Izquierdo, Rafael; Ahijado Hormigos, Francisco Hospital Virgen de la Salud (Toledo); Dotori, Marta. USP Marbella (Málaga); Duarte, Verónica. Hospital de Terrassa (Barcelona); Estupiñan Torres, Sara. Hospital Universitario Canarias (Santa Cruz de Tenerife); Fernández Reyes, Mª José. Hospital de Segovia (Segovia); Fernández Rodríguez, Mª Loreto. Hospital Príncipe de Asturias (Madrid); Fernández, Guillermina. Clínica Santa Isabel (Sevilla); Galán Serrano, Antonio. Hospital General Universitario de Valencia (Valencia); García Cantón, Cesar. Hospital Universitario Insular de Gran Canaria (Las Palmas); García Herrera, Antonio L. Hospital Universitario Puerto Real (Cádiz); García Mena, Mercedes. Hospital San Juan de Dios (Zaragoza); Gil Sacaluga, Luis; Aguilar, Maria. Hospital Virgen del Rocío (Sevilla); Górriz, José Luis. Hospital Universitario Doctor Peset (Valencia); Huarte Loza, Emma. Hospital San Pedro (Logroño); Lerma, José Luis. Hospital Universitario Salamanca (Salamanca); Liebana Cañada, Antonio. Hospital de Jaén (Jaén); Marín Álvarez, Jesús Pedro. Hospital San Pedro de Alcántara (Cáceres); Martín Alemany, Nàdia. Hospital Jose p Trueta (Girona); Martín García, Jesús. Hospital Nuestra Señora de Sonsoles (Ávila); Martínez Castelao, Alberto. Hospital Universitari de Bellvitge (Barcelona); Martínez Villaescusa, María. Complejo Hospitalario Universitario de Albacete (Albacete); Martínez, Isabel. Hospital Galdakao (Bilbao); Moina Eguren, Iñigo. Hospital Basurto (Bilbao); Moreno Los Huertos, Silvia. Hospital Santa Bárbara (Soria); Mouzo Mirco, Ricardo. Hospital El Bierzo, Ponferrada (León); Munar Vila, Antonia. Hospital Universitari Son Espases (Palma de Mallorca); Muñoz Díaz, Ana Beatriz. Hospital Virgen del Consuelo (Valencia); Navarro González, Juan F. Hospital Universitario Nuestra Señora de Candelaria (Santa Cruz de Tenerife); Nieto, Javier; Carreño, Agustín. Hospital General Universitario de Ciudad Real (Ciudad Real); Novoa Fernández, Enrique. Complexo Hospitalario de Ourense (Ourense); Ortiz, Alberto; Fernandez, Beatriz. IIS-Fundación Jiménez Díaz (Madrid); Paraíso, Vicente. Hospital Universitario del Henares (Madrid); Pérez Fontán, Miguel. Complejo Hospitalario Universitario A Coruña (A Coruña); Peris Domingo, Ana. Hospital Francesc de Borja (Valencia); Piñera Haces, Celestino. Hospital Universitario Marqués de Valdecilla (Santander); Prados Garrido, Mª Dolores. Hospital Universitario San Cecilio (Granada); Prieto Velasco, Mario. Hospital de León (León); Puig Marí, Carmina. Hospital d'Igualada (Barcelona); Rivera Gorrín, Maite. Hospital Universitario Ramón y Cajal (Madrid); Rubio, Esther. Hospital Puerta del Hierro (Madrid); Ruiz, Pilar. Hospital Sant Joan Despí Moisès Broggi (Barcelona); Salgueira Lazo, Mercedes; Martínez Puerto, Ana Isabel. Hospital Virgen Macarena (Sevilla); Sánchez Tomero, José Antonio. Hospital Universitario de la Princesa (Madrid); Sánchez, José Emilio. Hospital Universitario Central de Asturias (Oviedo); Sans Lorman, Ramon. Hospital de Figueres (Girona); Saracho, Ramon. Hospital de Santiago (Vitoria); Sarrias, Maria; Serón, Daniel. Hospital Universitari Vall d'Hebron (Barcelona); Soler, María José; Barrios, Clara. Hospital del Mar (Barcelona); Sousa, Fernando. Hospital Rio Carrión (Palencia); Toran, Daniel. Hospital General de Jerez (Cadiz); Tornero Molina, Fernando. Hospital de Sureste (Arganda del Rey); Usón Carrasco, José Javier. Hospital Virgen de la Luz (Cuenca); Valera Cortes, Ildefonso. Hospital Virgen de la Victoria (Málaga); Vilaprinyo del Perugia, Mª Merce. Institut Catala d'Urologia i Nefrologia (Barcelona); Virto Ruiz, Rafael C. Hospital San Jorge (Huesca); Vicente Pallarés Carratalá Clinica MEDEFIS (Vila-real. Castellón), Carlos Santos Altozano CS Azuqueca de Henares (Guadalajara); Miguel Artigao Ródenas CS Zona III (Albacete); Inés Gil Gil Área Básica Sanitaria de Arán. CAP Viella (Lleida); Francisco Adan Gil CS Alfaro (La Rioja); Emilio García Criado Centro de Salud del Carpio (Córdoba); Rafael Durá Belinchón CS Godella (Valencia); Jose Mª Fernández Toro CS Zona Centro (Cáceres); Juan Antonio Divisón Garrote Centro de Salud de Casas Ibáñez. Consultorio de Fuentealbilla (Albacete).
