# Supplementary material for: Matrix Gla protein polymorphism rs1800802 is associated with atheroma plaque progression and with cardiovascular events in a chronic kidney disease cohort
Source: Clin Kidney J. 2023 Oct 10;17(1):sfad257. doi: 10.1093/ckj/sfad257 (PMC10768782; doi:10.1093/ckj/sfad257)
Supplement: sfad257_Supplemental_File [file sfad257_supplemental_file.docx]

| Supplemental Table 1: Association of MGP rs1800802 T/C polymorphism to epidemiological, clinical, biochemical parameters and MGP levels. | | | | | |
| --- | --- | --- | --- | --- | --- |
| Variable | | | Homozygote TT + Heterocygote CT  n=48 | Homozygote CC  n=32 | p |
| Age (years) | | | 65[59;70] | 62.5[54;70] | 0.225 |
| Sex (female) | | | 15(31.3) | 8(34.8) | 0.545 |
| Smoking | | | 33(68.8) | 26(81.3) | 0.213 |
| Diabetes | | | 19(39.6) | 13(40.6) | 0.926 |
| Hypertension | | | 44(91.7) | 29(90.6) | 0.872 |
| Dyslipemia | | | 40(83.3) | 27(84.4) | 0.902 |
| CKD Stage | | G3 | 25(25.1) | 15(46.9) | 0.877 |
|  | | G4-5 | 17(35.4) | 12(37.5) |  |
|  |  | G5D | 6(12.5) | 5(15.6) |  |
| Body mass index (kg/m^2^) | | | 29[26;33.3] | 28[24.8;31.6] | 0.321 |
| Systolic Blood Pressure (mmHg) | | | 141.5[133;155] | 141[128;160] | 0.996 |
| Diastolic Blood Pressure (mmHg) | | | 81[74;87] | 80.5[70;88.5] | 0.848 |
| Pulse Pressure (mmHg) | | | 63[53;71] | 57.5[49;72] | 0.662 |
| Total Cholesterol (mg/dL) | | | 178[162;194] | 174.5[141;203.5] | 0.678 |
| HDL Cholesterol (mg/dL) | | | 46.5[41;52] | 47[37;50.5] | 0.573 |
| LDL Cholesterol (mg/dL) | | | 102[80;119] | 96[74.7;112.6] | 0.746 |
| Triglycerides (mg/dL) | | | 164[110;235] | 130[91;195] | 0.191 |
| Glucose(mg/dL) | | | 103[91;119] | 111[89;151] | 0.763 |
| Calcium (mg/dL) | | | 9.3[9;9.6] | 9.2[9;9.5] | 0.847 |
| Phosphate (mg/dL) | | | 3.6[3.2;4.2] | 3.75[3.1;4] | 0.859 |
| Sodium (mEq/L) | | | 141[139;142] | 139[137;141] | 0.069 |
| Potassium (mEq/L) | | | 4.9[4.4;5.2] | 4.7[4.2;5.4] | 0.698 |
| 25 OH D (ng/ml) | | | 13.7[10.4;16.3] | 16.28[11.7;21.58] | 0.114 |
| Plaque at baseline | | | 44(91.7) | 27(84.4) | 0.312 |
| Plaque progression | No progression | | 17(35.4) | 6(18.8) | 0.23 |
|  | 1-2 plaques | | 19(39.6) | 14(43.8) |  |
|  | ≥3 plaques | | 12(25) | 12(37.5) |  |
| Cardiovascular Event | | | 2(4.2) | 6(18.8) | 0.033 |
| Total MGP (pg/ml) | | | 275[140;730] | 414[171;709] | 0.346 |
| dp-ucMGP (pg/ml) | | | 427[315;657] | 428[330;622] | 0.806 |
| Qualitative variables are expressed as N (%). Quantitative variables are expressed as median [Q1;Q3]. Comparisons between groups was performed with Mann-Whitney U test for quantitative variables, and Chi-squared test for categorical data.  CKD: Chronic kidney disease, HDL: High-density lipoprotein, LDL: Low-density lipoprotein,25 OH: 25 hydroxy vitamin D, MGP: Matrix Gla-Protein, dp-ucMGP: uncarboxilated and dephophorilated MGP. | | | | | |

| Supplemental Table 2: Association of MGP levels with rs1800802 polymorphism | | | | |
| --- | --- | --- | --- | --- |
| Genotype | Total MGP | | dp-ucMGP | |
|  | Protein levels (pg/ml) | p | Protein levels (pg/ml) | p |
| TT (31) | 396[144;962] | 0.069 | 461[366;677] | 0.131 |
| CT (17) | 209[123;307] |  | 343[296;580] |  |
| CC (32) | 414[171;709] |  | 429[330;622] |  |
| Quantitative variables are expressed as median [Q1; Q3]. Comparisons between groups was performed with Kruskal-Wallis H Test.  dp-ucMGP: Dephosphorylated-uncarboxylated –MGP protein | | | | |

| Supplemental Table 3: Association of MGP levels with plaque presence, progression and cardiovascular events | | | |
| --- | --- | --- | --- |
| Variable | Total MGP levels (pg/ml) | | p |
|  | No | Yes |  |
| Any Basal Plaque | 387[110;783] | 333[164;725] | 0.825 |
| ≥1 new plaque | 274[144;432] | 395[157;775] | 0.285 |
| ≥3 new plaques | 326[140;737] | 380[161;598] | 0.891 |
| Cardiovascular Event | 286[140;656] | 722[397;949] | 0.034 |
| Quantitative variables are expressed as median [Q1; Q3]. Comparisons between groups was performed with Mann-Whitney U test.  Any Basal Plaque (No:9; Yes:71),≥1 new plaque (No:23; Yes:57), ≥3 new plaques (No:56; Yes:24 ) ECV (No:72; Yes:8) | | | |

| Supplemental Table 4: Association of Dephosphorylated-uncarboxylated -MGP levels with plaque presence, progression and cardiovascular events | | | |
| --- | --- | --- | --- |
| Variable | Total dp-ucMGP levels (pg/ml) | | p |
|  | No | Yes |  |
| Any Basal Plaque | 436[333;923] | 419[319;624] | 0.573 |
| ≥1 new plaque | 419[302;672] | 434[338;636] | 0.401 |
| ≥3 new plaques | 420[317;669] | 444[326;602] | 0.721 |
| Cardiovascular Event | 418[314;642] | 514[409;639] | 0.191 |
| Quantitative variables are expressed as median [Q1; Q3]. Comparisons between groups was performed with Mann-Whitney U test.  Any Basal Plaque (No:9; Yes:71),≥1 new plaque (No:23; Yes:57), ≥3 new plaques (No:56; Yes:24 ) ECV (No:72; Yes:8) | | | |
